# Impact of physical confinement on nuclei geometry and cell division dynamics in 3D spheroids

**DOI:** 10.1038/s41598-018-27060-6

**Published:** 2018-06-08

**Authors:** Annaïck Desmaison, Ludivine Guillaume, Sarah Triclin, Pierre Weiss, Bernard Ducommun, Valérie Lobjois

**Affiliations:** 1 ITAV, Université de Toulouse, CNRS, Toulouse, France; 2 IMT, Université de Toulouse, CNRS, Toulouse, France; 3 CHU de Toulouse, Toulouse, France

## Abstract

Multicellular tumour spheroids are used as a culture model to reproduce the 3D architecture, proliferation gradient and cell interactions of a tumour micro-domain. However, their 3D characterization at the cell scale remains challenging due to size and cell density issues. In this study, we developed a methodology based on 3D light sheet fluorescence microscopy (LSFM) image analysis and convex hull calculation that allows characterizing the 3D shape and orientation of cell nuclei relative to the spheroid surface. By using this technique and optically cleared spheroids, we found that in freely growing spheroids, nuclei display an elongated shape and are preferentially oriented parallel to the spheroid surface. This geometry is lost when spheroids are grown in conditions of physical confinement. Live 3D LSFM analysis of cell division revealed that confined growth also altered the preferential cell division axis orientation parallel to the spheroid surface and induced prometaphase delay. These results provide key information and parameters that help understanding the impact of physical confinement on cell proliferation within tumour micro-domains.

## Introduction

Multicellular spheroids reproduce the three-dimensional (3D) multicellular architecture, cell-cell interactions as well as oxygen and proliferation gradients observed in tumour micro-domains^1,2^. Multicellular spheroids have been used as surrogate to investigate the consequence of the resistance opposed by the tumour microenvironment to tumour growth *in vivo*. When multicellular aggregates are grown in constrained conditions, for instance upon embedding within agarose gel, alginate capsules or polydimethylsiloxane (PDMS) pillars^3–7^, growth is altered, cell proliferation is reduced and apoptosis is increased^5,7,8^. Growth confinement might also alter cell shape at the spheroid periphery^9^. In a previous study, we explored the effect of a confined environment on spheroid growth, and showed that multicellular aggregates grown in PDMS micro-channels for several days adopt an elongated rod-like shape with the longer axis parallel to the channel axis^10^. This growth directionality is accompanied by accumulation of mitotic cells within spheroids. This accumulation is not associated with alteration of cell rounding, but with spindle pole abnormalities^10^.

Most of the studies on the impact of confinement on spheroid growth and proliferation were based on the analysis of the spheroid global shape, or on the characterization at the cell scale using imaging strategies based on 2D optical sections or cryosections. However, these approaches are not appropriate to characterize the impact of confinement on cell behaviour parameters, such as shape and orientation of nuclei, as well as the orientation of cell division that should be performed directly using 3D data.

To address this challenge we used light-sheet fluorescence microscopy (LSFM) to acquire 3D images of optically cleared spheroids and to monitor cell division in live spheroids^11,12^. Moreover, we developed dedicated algorithms and software tools for semi-supervised 3D image analysis. We found that, in spheroids grown in suspension, nuclei have an elongated shape and are preferentially oriented parallel to the spheroid surface, like the cell division axes. These features were altered in spheroids grown in confined conditions following embedding in low-melting point agarose. Live monitoring of cell division within spheroids grown in constrained and non-constrained conditions revealed that prometaphase was delayed in spheroids embedded in low-melting point agarose.

These results are consistent with the growing body of evidence that the physical microenvironment is an essential actor of tumour growth, and suggest that confinement could impair cell division and thus, might contribute to genomic instability.

## Results

### 3D analysis of the geometry of cell nuclei in spheroids

**Figure 1:**
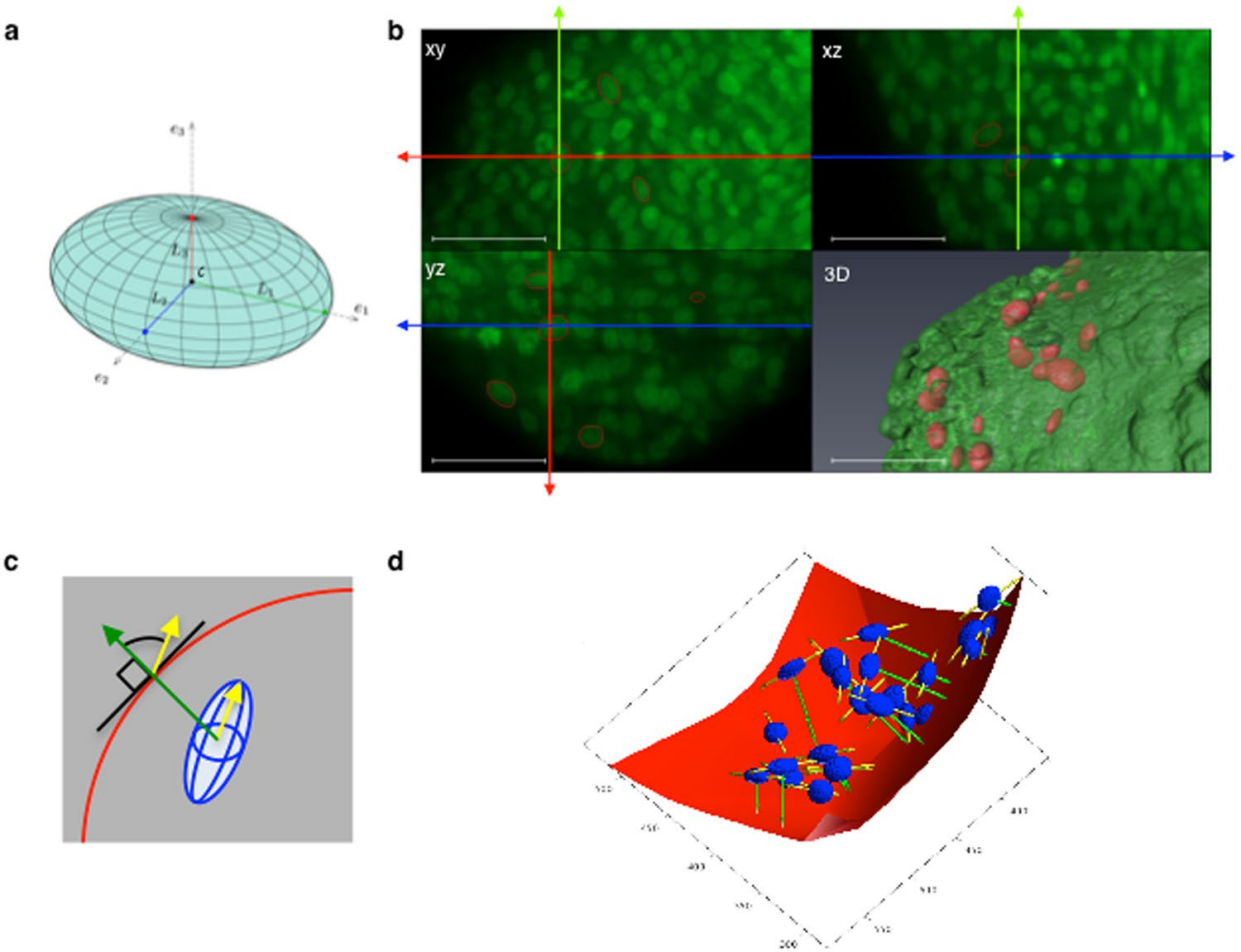
Principle of the determination of 3D nuclei geometry. Spheroids were fixed and stained with propidium iodide (a fluorescent nuclear marker). After optical clearing through ethanol dehydration and incubation in an organic solvent (BABB), nuclei in the whole spheroid volume were imaged by light-sheet fluorescence microscopy. (**a**) Geometrical parameters of an ellipsoid. (**b**) Images of the xy, xz and yz planes of a portion of a z-stack with propidium iodide fluorescence shown in green. The red ellipses show the result of the 3D segmentation of nuclei performed using the FitEllipsoid Icy 3D segmentation plugin in each plane. Green, red and blue axes indicate the position in x, y and z respectively. The 3D visualization (lower right panel) corresponds to the volume rendering of a LSFM spheroid z-stack (AMIRA software) in green with the 3D iso-surfaces of segmented nuclei in red. Scale bar: 50 µm. (**c**) The orientation of a nucleus (in blue) is defined as the angle of the long axis (yellow arrow) relative to the normal (in green) at the closest point on the spheroid surface (in red). (**d**) 3D visualization of the spheroid convex hull (red) and the ellipsoids corresponding to the nuclei (blue) from a z-stack. The direction of the long axis and the closest point on the spheroid convex hull are shown in yellow and green, respectively.

Previous studies performed using spheroid cryosections suggest that nuclei located at the spheroid periphery tend to be elongated and aligned with the spheroid boundary^13^. However, in spheroid sections, nuclei are randomly cut and information on their shape can only be very partial. A more accurate characterization requires determining their 3D geometry. The nucleus shape can be described using ellipsoids that are defined by a centre c, three orthogonal unit vectors denoted e1, e2 and e3 in R^3^, and their respective lengths L1, L2 and L3 (Fig. 1a). Therefore, our objective was to develop a method to fit ellipsoids in a 3D environment to mimic nuclei within spheroids. We previously reported that light-sheet fluorescence microscopy (LSFM) is well suited to perform 3D imaging of large samples, such as spheroids derived from HCT116 cancer cells^11,12,14^. Optical clearing with organic solvents efficiently improves in-depth 3D imaging of thick samples by LSFM^15^. Here, we adapted clearing with benzyl alcohol-benzyl benzoate (BABB) to spheroids stained with propidium iodide after fixation to improve in depth resolution for the analysis of the 3D shape of nuclei (Fig. 1b). The automatic segmentation of nuclei in 3D LSFM images was not possible using open-source or commercial software tools that were not efficient enough to segment large collections of nuclei from a huge dataset, such as in LSFM z-stacks of spheroids. Therefore, we designed a specific semi-supervised algorithm to efficiently fit ellipsoids in 3D to nuclei (Fig. 1b) and to extract the c, e1, e2 and e3 coordinates as well as the L1, L2 and L3 lengths. The algorithm is available and distributed as a plugin for Icy, a widely used open community platform for bioimage informatics (https://www.youtube.com/watch?v=MjotgTZi6RQ&feature=youtu.be)^16^.We determined the nucleus elongation on the basis of its aspect ratio defined as the ratio between its longest axis L1 and its shortest axis L3.

**Figure 2:**
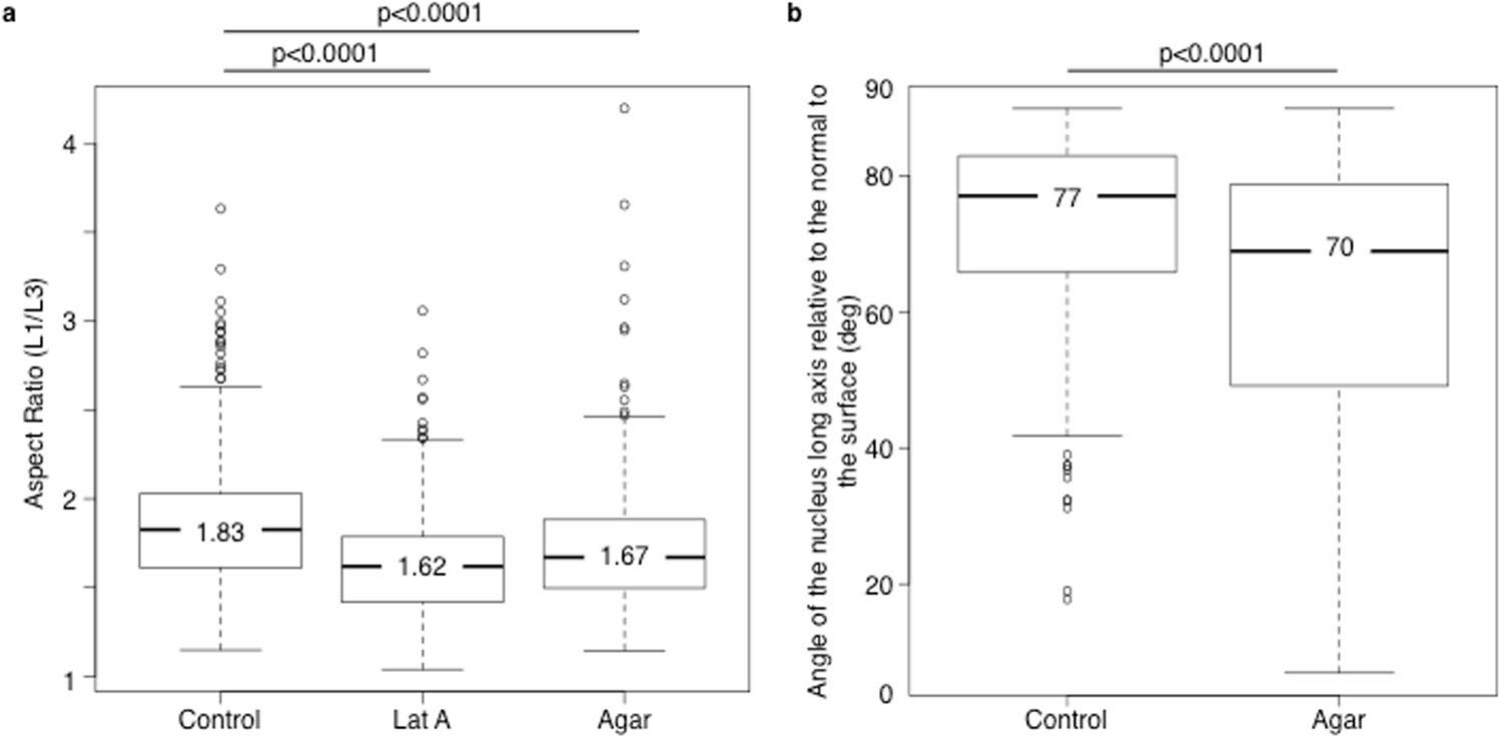
Analysis of nucleus geometry in spheroids. (**a**) Boxplots (R software) of the L1 to L3 ratio in control spheroids (control), spheroids incubated with latrunculin A (500 nM) for 8 hours (Lat A) or grown in 1% low-melting point agarose for 24 hours (Agar) before fixation. A high ratio value indicates that the nucleus is elongated; n = 272, 244 and 429 nuclei from control, latrunculin A-treated and agarose-embedded spheroids, respectively. 6 to 13 independent spheroids were analysed in each condition. (**b**) Boxplots showing the orientation of nuclei in control and agarose-embedded (Agar) spheroids. Only nuclei with an L1/L3 value higher than 1.5 (and thus considered to be elongated) were analysed. A 90° angle means that L1 is parallel to the spheroid convex hull. In control, the nucleus angle values are significantly different from those in agarose-embedded spheroids (p < 0.0001 in both case).

Using this methodology, we found that nuclei located between 0 and 100 µm from the surface in spheroids grown in suspension had an elongated shape (median aspect ratio = 1.83) (Fig. 2a, Fig. S1). As a control, we showed that, in agreement with our previous results based on cryosections^13^, nuclei of spheroids incubated with latrunculin A, a pharmacological agent that prevents actin polymerization, were rounder (median aspect ratio = 1.62) (Fig. 2a).

Considering the elongated shape of nuclei, we then analysed their orientation relative to the spheroid surface. From a mathematical viewpoint, the nucleus orientation parallel to the spheroid surface means that the vector e1, which is collinear with the nucleus longest axis, approximately belongs to the plane tangent to the spheroid surface at the point nearest to the ellipsoid centre c (Fig. 1c). To find this point for each fitted ellipsoid, we generated the convex hull of the whole spheroid volume from 3D images and used it as a mesh for the spheroid surface (Fig. 1d). Then, for each segmented nucleus, we found the point on the convex hull nearest to the centre of the corresponding ellipsoid and the plane tangent to the surface at this point. Using this experimental approach, we measured the angle between the ellipsoid main axis and the normal to the tangent plane, and we found that, for elongated nuclei with an aspect ratio higher than 1.5 (87% of all nuclei), the median angle value was 77°, suggesting that nuclei were oriented quite parallel to the spheroid surface (Fig. 2b).

### 3D analysis of cell division axis orientation within spheroids

As our findings indicated that nuclei between 0 and 100 µm from the surface are preferentially elongated and orientated parallel to the spheroid surface, we asked whether the cell division axis orientation was also parallel to the spheroid surface.

**Figure 3:**
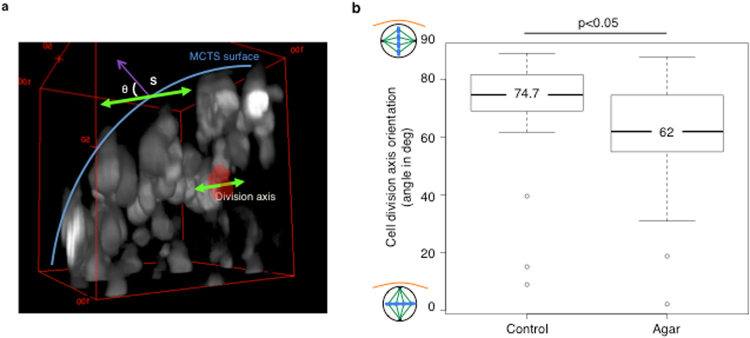
Analysis of the cell division axis orientation. Mitotic cells in the z-stack of a spheroid are detected based on the chromatin condensation visualized thanks to histone H2B-mCherry fluorescence (HCT116-H2B-mCherry cells). (**a**) 3D visualization of a portion of one HCT116-H2B-mCherry cell spheroid from a LSFM acquisition z-stack. The red surface delineates the metaphase plate. The green arrows indicate the 3D orientation of the division axis. The blue line shows the spheroid surface. The orientation of the division axis was determined by measuring the angle of the division axis relative to the normal to the spheroid surface (MCTS) at the nearest point (S, purple arrow). An angle of 90° corresponds to a division axis parallel to the spheroid surface and a metaphase plate perpendicular to the surface. (**b**) Boxplots of the angle of the division axis in control and in low-melting point agarose-embedded spheroids.

The cell division axis is defined as the axis perpendicular to the metaphase plate. As the probability to detect a mitotic cell in the plane of the section is very low in spheroid cryosections, we performed this analysis using 3D images. To optimize the detection of metaphases, we monitored mitotic cells by time-lapse LSFM in spheroids made of HCT116 cells that express a histone H2B-mCherry fusion protein (Supplementary Movie 1). The achieved image resolution allowed us to distinguish metaphase plates (see Supplementary Movie 2). To determine the division axis orientation, we developed a procedure described in details in the Methods section. Briefly, the metaphase plate is segmented and represented as a 3D surface by a thin and homogeneous ellipsoid, flattened along one axis (Fig. 3a) and the descriptive parameters (the x, y and z coordinates of the mass centre and the x, y and z coordinates of the three orthogonal axes) are extracted. Then, the orientation of the division axis is determined by calculating the angle of the shortest axis of the ellipsoid with reference to the normal to the tangent at the closest point to the spheroid convex hull (Fig. 3a). Using this method, we observed that, in freely grown spheroids, the division axis was preferentially parallel to the surface (median angle = 74.7°) in most mitotic cells (Fig. 3b).

### Physical confinement alters the 3D nucleus geometry and cell division axis orientation

To investigate the impact of mechanical confinement on the 3D nucleus geometry and cell division axis orientation, we used the previously described experimental strategy and spheroids grown in 1% low-melting point agarose for 24 hours. In this condition, it has been shown that stress accumulates around spheroids due to displacement of the gel by the growing spheroid^5,6^. After embedding, spheroids were in contact with the surrounding agarose (Fig. S2) and after around 30 hours, they ruptured the gel, leading to a rapid deformation of the spheroid shape. This indicates that agarose limits the spheroid growth (Fig. S2 and Supplementary Movie 3).

Compared with spheroids grown in suspension (control), nuclei located between 0 and 100 µm from the spheroid surface were significantly less elongated in spheroids grown in agarose (median aspect ratio = 1.67, p < 0.0001 compared with control) (Fig. 2a). Moreover, elongated nuclei (75% of all nuclei) were less parallel to the spheroid surface (median angle = 70°, p < 0.0001 compared with control) (Fig. 2b), and the preferential orientation of the cell division axis was changed (median angle = 62°, p < 0.05 compared with control) (Fig. 3b). Conversely, the geometry of nuclei deeper than 100 µm within the spheroid was similar in control and confined spheroids (Fig. S1). Together, these results indicate that confined growth conditions alter the 3D nuclei geometry and the cell division axis orientation only in the outmost cell layers of spheroids.

### 3D cell division dynamic monitoring reveals prometaphase delay upon constrained growth

**Figure 4:**
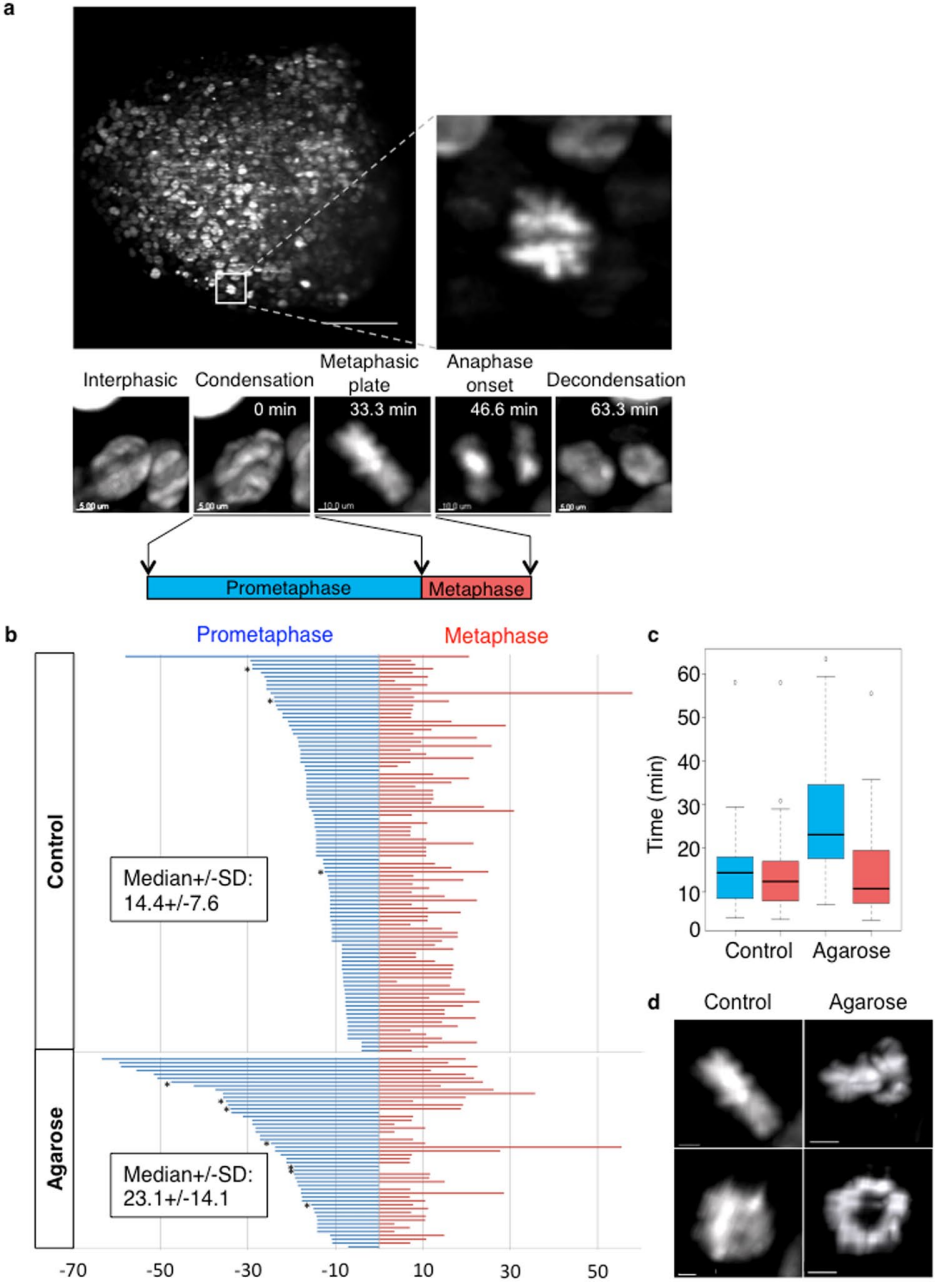
3D cell division dynamic monitoring reveals longer prometaphase duration in spheroids grown in agarose. (**a**) 3D time-lapse imaging of cell progression through mitosis using LSFM allows monitoring the cell division dynamics. Top panel: maximal projection of a z-stack (left) and zoom on a mitotic cell (right). Scale bar: 100 µm. Bottom panels: selected in depth images of cells at the indicated stages of mitosis. Time after nuclear condensation is indicated. Prometaphase duration: time between DNA condensation and metaphase initiation. Metaphase duration: time between the establishment of the metaphase plate and anaphase initiation. (**b**) Prometaphase (blue) and metaphase (red) duration (minutes) measured in mitotic cells within control (n = 98) and agarose-embedded spheroids (n = 50). Each bar represents the duration of these two phases in one mitotic cell in which prometaphase and metaphase were fully monitored (98 out of 104 cells in control, and 50 out of 78 cells in agarose-embedded spheroids). Stars indicate mitotic cells where condensation started before the beginning of the time-lapse video recording. Consequently, in these cells, prometaphase was longer than the duration indicated in the graph. All cells were aligned relative to the metaphase plate. The median (±SD) duration is indicated. (**c**) Boxplot of the prometaphase (blue) and metaphase (red) duration in control and agarose-embedded spheroids. (**d**) (Left panels): 3D images of a mitotic cell in a control spheroid with chromosomes organized in a metaphase plate. Scale bar: 5 µm. (Right panels): 3D images of a mitotic cell in a spheroid grown in agarose with chromosomes organized in a ring. Scale bar: 10 µm. Side view (upper panels) and front view (lower panels) are shown.

Cell division is largely dependent on the cell and nucleus geometry during interphase^17–20^ and their alteration can affect mitosis progression^21,22^. Therefore, we examined the impact of confinement on progression into mitosis. To this aim, we performed live LSFM imaging to monitor cell division in HCT116 cell spheroids that express histone H2B-mCherry (Supplementary Movie 3, Fig. 4a). From LFSM time lapse experiments, the image resolution (Fig. 4a) allowed us to precisely identify the steps of cell division and the time point at which they occur: DNA condensation, alignment of chromosomes on the metaphase plate, and separation of chromosomes at anaphase onset. From these data, we could calculate the duration of prometaphase (*i.e*., the time interval between DNA condensation and metaphase plate formation) and of metaphase (*i.e*., the time interval between metaphase plate formation and anaphase onset). Anaphase duration corresponds to the time between anaphase onset and the beginning of DNA decondensation and cytokinesis, and is much more difficult to identify. For this reason, we determined only the prometaphase and metaphase duration in a large number of dividing cells within spheroids grown in suspension (control, Supplementary Movie 4) or in 1% low-melting point agarose for 24 h (Supplementary Movie 5). In control spheroids, prometaphase lasted on average 14.4 ± 7.6 minutes and metaphase 13 ± 5 minutes. Growth in agarose increased prometaphase duration (23.1 ± 14.1 minutes, p < 0.0001 compared with control), but did not affect metaphase duration (Fig. 4b,c). When we considered also mitotic cells the metaphase plate of which could not be detected in the recorded movies (not included in Fig. 4b), we found that 22% of mitotic cells in spheroids grown in agarose had a prometaphase longer than 40 minutes, but only 1% in control spheroids. Moreover, in mitotic cells with longer prometaphase, condensed chromosomes were organized as a persistent ring with a central lumen instead of the full cylinder that corresponds to the metaphase plate observed in controls (Fig. 4d and Supplementary Movies 6 and 7). This ring-shaped organization of the chromosomes could correspond to the structure that has been described for the capture of chromosomes by microtubules during prometaphase^23^.

## Discussion

Biochemical and biomechanical cues contribute to tumour development. Particularly, it has been shown that stiffening of the extracellular matrix during tumour growth contributes to the loss of tensional homeostasis in solid tumours^24,25^. Moreover, the extracellular matrix in the tumour microenvironment limits tumour expansion and imposes a mechanical stress. Spheroids embedded within an inert biocompatible matrix made of agarose have been used to demonstrate the accumulation of mechanical stress in constrained growth conditions. In such constrained conditions, cell proliferation also is decreased^5,6^. In a previous study, we showed that constrained growth induces accumulation of mitotic cells within multicellular tumour spheroids. The objective of the present study was to analyse the impact of mechanical confinement on tumour cell division dynamics in relation with the 3D cell organization. To this aim we developed a 3D methodology and used spheroids embedded in agarose. This cell culture method allows considering only the impact of the mechanical resistance from the microenvironment on the organization of cells within spheroids and on cell division orientation and dynamics, independently of signalling pathways. We developed an interdisciplinary approach combining 3D fluorescence live imaging technology and proprietary software to quantitatively analyse these parameters. LSFM implementation has been a major breakthrough for the imaging of large and scattering 3D models, such as spheroids^14,26^. Using cell lines that express fluorescent histone H2B and specifically designed sample holders, we previously showed that this technology allows monitoring live cell division dynamics in large 3D spheroidal micro-tissues^12^. However, despite this 3D imaging improvement, data quality and size were still not compatible with fully automated segmentation procedures. Therefore, we developed dedicated algorithms and software tools that demonstrate the advantage of using semi-supervised algorithms for 3D image analyses compared with time-consuming fully manual segmentation strategies.

Here, using this methodology, we show that nuclei display a preferential 3D orientation and elongation parallel to the spheroid surface. The analysis of cell shape in spheroids also indicates that cells tend to be elongated along the spheroid surface^9^. We also found that the cell division axis is preferentially oriented parallel to the spheroid surface and therefore to the nucleus elongation axis. Altogether, these data suggest that the spheroid cell organization could be dependent on mechanical strain relative to the spheroid surface that induces cell and nuclei elongation and orientation parallel to the maximal strain axis.

Several studies have shown that in cultured adherent cells and in developing tissues, the cell division axis is oriented along the axis of maximal tension^27–29^. In cells grown on adhesive patterns, focal adhesions and actin cytoskeleton produce internal cortical forces that direct the orientation of the division axis^30^. Cell shape also guides spindle orientation. In cell monolayers under stretch, cell division is mostly aligned along the interphase long axis^31^. It has been shown in flies and plants that cells adjust cell division orientation to their shape following mechanical strain^32^. Similarly, in sea urchin embryos, cell shape dictates the positioning and orientation of the nucleus and division axis through pulling forces exerted by microtubules^33^ and in endothelial cells cultured on adhesive micro-patterns, cell elongation alters the nucleus shape and orientation relative to the cell long axis^20^.

It has been already reported that spheroid growth in conditions of confinement induces cell cycle arrest in the G1-phase^8^. Our work shows that in such conditions, mitosis progression also is altered. Indeed, in physically confined growth conditions, we observed that prometaphase duration was increased. This could results from an alteration of spindle positioning induced by the modification of mechanical strain and nuclei organization. We could not test this hypothesis by live 3D microscopy because of the alteration of the global morphology of spheroids of HCT116 cell engineered to express tubulin-GFP (data not show), and the insufficient resolution for 3D reconstruction of the mitotic spindle after live LSFM imaging of spheroids. However, the ring-shaped condensed chromosome organization observed in cells with extended prometaphase within spheroids grown in agarose is similar to the chromosome arrangement in the equatorial ring in cultured human cells. This structure is associated with transient unstable interactions between kinetochores and microtubules during spindle assembly in early prometaphase^23^. Therefore, our observations strongly suggest that in conditions of physical confinement, mitotic spindle assembly and/or positioning is transiently impaired, leading to activation of the spindle assembly checkpoint^34,35^, and increased prometaphase duration. Spindle assembly and orientation are crucial for chromosome capture and segregation, and consequently for tissue organization and development^27,36,37^. Spindle misorientation alone is unlikely to be tumorigenic^38^; however, spindle defects contribute to carcinogenesis, suggesting that they synergize with cancer-associated mutations and promote genomic instability^37^.

## Methods

### Cell culture and spheroid generation

HCT116 colorectal adenocarcinoma cells (ATCC, nearly diploid and Ras mutated) that express histone H2B fused with mCherry were obtained by lentiviral transfection. Cells were cultured in DMEM (Gibco) containing 10% foetal calf serum with 2 mM/l glutamine and penicillin/streptomycin in a humidified atmosphere of 5% CO_2_ at 37 °C. In these culture conditions, HCT116 cells have an epithelial morphology. Spheroids were prepared as previously described^39^. Briefly, 500 cells/well were distributed in poly-HEMA-coated 96-well round bottom plates. Plates were centrifuged (300 g for 6 min) and then placed in a humidified atmosphere of 5% CO_2_ at 37 °C. Spheroids of 650 µm in diameter were kept in suspension, incubated or not with 0.5 µM latrunculin A for 8 hours, or transferred in 1% Low-Melting Point agarose (10 g/L, Euromedex) for 24 hours before further analysis.

### Light-sheet fluorescence microscopy of optically cleared spheroids

HCT116 cell spheroids were fixed at room temperature with formalin (Sigma) for 4 hours. Spheroids were stained with propidium iodide, embedded in an agarose cylinder, and then sequentially transferred in 25%, 50%, 75% and finally 95% ethanol solutions for complete dehydration. For clearing, samples were transferred in BABB solution (1:2 benzyl alcohol: benzyl benzoate, Sigma). Spheroids immersed in BABB were then imaged by LSFM^25^ using a 10x objective. The whole volume of each spheroid was imaged with a 1 µm z-step.

### Nucleus geometry analysis

The FitEllipsoid plugin for Icy was used^16,40^. Briefly, from the propidium iodide fluorescence signal, a few points on the nucleus boundary were manually delimited on the xy, yz and xz views on a central z-plane of each nucleus analysed. Then, an ellipsoid was fitted automatically to the set of selected points. The ellipsoid geometrical parameters were then saved by the plugin. A MATLAB algorithm was used to determine the geometry of nuclei within the spheroid from this data. The user provides the image stack of the spheroid and a file with the parameters of all the identified ellipsoids (nuclei). The algorithm first identifies the spheroid boundary from the stack of images and extracts the convex hull. Then, for each ellipsoid it finds the closest point on the envelope, and finally calculates the angle between the ellipsoid longest axis and the normal to the surface. Uneven fluorescent signal intensity in nuclei and non-homogeneous imaging of the whole spheroid might preclude the envelope extraction. To address this potential limitation, the following steps are undertaken by the algorithm. First, the image is denoised by a Gaussian convolution. By thresholding, a 3D binary image that roughly corresponds to the spheroid is obtained, and then the spheroid form is regularized by finding the smallest convex polyhedron that contains all the points of the binary image. The output is a triangulation of the envelope. To detect the closest point of a nucleus to the surface, the projection of the ellipsoid centre is calculated on all the envelope triangles and the shortest distance is kept.

### Live light-sheet fluorescence microscopy imaging of spheroids

Samples were prepared as previously described^11^. Briefly, sample chambers in hydrogel (Phytagel, 10 g/L in PBS) were fabricated and filled with culture medium or melted agarose. Spheroids were transferred into the chambers 24 hours prior acquisition. LSFM setup, 3D and time-lapse image acquisition were as previously specified^12^. Chambers were hanged with a rod and immersed in medium in the physiologic chamber. Image stacks of 170 to 300 µm from the spheroid periphery to the centre, with 1 µm step, were acquired every 4 minutes for a maximum of 4 hours.

### Analysis of the cell division orientation axis

Mitotic cells were visualized in 3D fluorescence images acquired by LSFM using the Imaris software. The metaphase plate was segmented and represented as an ellipsoid flattened along one axis that indicates the division axis orientation. The ellipsoid descriptive parameters (the x, y and z coordinates of the centre of the mass and the x, y and z coordinates of the three orthogonal axes) corresponding to the segmented object were then extracted. The last metaphase plate detected before anaphase onset was used to determine the shortest axis of the ellipsoid that corresponds to the division axis orientation. This orientation refers to a coordinate reference that is the normal to the spheroid surface at the closest point. To obtain this reference, a MATLAB program was used to determine the spheroid convex hull and calculate the distance of mitotic cells from the surface. The angle of the ellipsoid shortest axis with the normal to the surface was then calculated to obtain the orientation of the division axis.

### Analysis of the duration of mitotic steps

Stack images were downloaded with Imaris to allow their 3D visualization. For each detected dividing cell, DNA condensation was determined as the first frame where the nucleus appears non-homogeneous. The metaphase plate corresponds to the thinner, full and homogeneous cylinder of chromosomes. Anaphase corresponds to the first frame in which the separation of the sister chromatids is detected. The time measured between DNA condensation and metaphase corresponds to the prometaphase duration. The time measured between the establishment of the metaphase plate and anaphase corresponds to the metaphase duration.

### Data analysis

Graphs and data were analysed with GraphPad Prism version 6.00, GraphPad software, La Jolla California USA, www.graphpad.com and in R (R Core Team 2017, https://www.R-project.org/). The non-parametric Mann-Whitney test was used to compare data from different conditions. Stacks were analysed using the Amira, Imaris and Fiji^41^ software programs.

## Electronic supplementary material

Supplementary information

Supplementary movie 1: Live 3D light-sheet fluorescence microscopy (LSFM) imaging of a spheroid.

Supplementary movie 2: Live 3D light-sheet fluorescence microscopy (LSFM) imaging of a mitotic cell in a spheroid.

Supplementary movie 3: Live imaging of an HCT116 spheroid after confinement in agarose.

Supplementary movie 4: Live imaging of the mitosis steps in a spheroid.

Supplementary movie 5: Live imaging of mitosis in an agarose-confined spheroid

Supplementary movie 6: Metaphase plate in a spheroid

Supplementary movie 7: Metaphase plate in a confined spheroid

## References

[1] Costa EC (2016). 3D tumor spheroids: an overview on the tools and techniques used for their analysis. Biotechnology advances.

[2] Lobjois V, Frongia C, Jozan S, Truchet I, Valette A (2009). Cell cycle and apoptotic effects of SAHA are regulated by the cellular microenvironment in HCT116 multicellular tumour spheroids. Eur J Cancer.

[3] Alessandri K (2013). Cellular capsules as a tool for multicellular spheroid production and for investigating the mechanics of tumor progression *in vitro*. Proc Natl Acad Sci USA.

[4] Aoun L (2014). Microdevice arrays of high aspect ratio poly(dimethylsiloxane) pillars for the investigation of multicellular tumour spheroid mechanical properties. Lab on a chip.

[5] Cheng G, Tse J, Jain RK, Munn LL (2009). Micro-environmental mechanical stress controls tumor spheroid size and morphology by suppressing proliferation and inducing apoptosis in cancer cells. PLoS One.

[6] Helmlinger G, Netti PA, Lichtenbeld HC, Melder RJ, Jain RK (1997). Solid stress inhibits the growth of multicellular tumor spheroids. Nature biotechnology.

[7] Montel F (2011). Stress clamp experiments on multicellular tumor spheroids. Physical review letters.

[8] Delarue M (2014). Compressive stress inhibits proliferation in tumor spheroids through a volume limitation. Biophysical journal.

[9] Dolega ME (2017). Cell-like pressure sensors reveal increase of mechanical stress towards the core of multicellular spheroids under compression. Nature communications.

[10] Desmaison A, Frongia C, Grenier K, Ducommun B, Lobjois V (2013). Mechanical stress impairs mitosis progression in multi-cellular tumor spheroids. PLoS One.

[11] Desmaison A, Lorenzo C, Rouquette J, Ducommun B, Lobjois V (2013). A versatile sample holder for single plane illumination microscopy. Journal of microscopy.

[12] Lorenzo C (2011). Live cell division dynamics monitoring in 3D large spheroid tumor models using light sheet microscopy. Cell Div.

[13] Zhang W (2016). Structure Tensor Based Analysis of Cells and Nuclei Organization in Tissues. IEEE transactions on medical imaging.

[14] Pampaloni F, Reynaud EG, Stelzer EH (2007). The third dimension bridges the gap between cell culture and live tissue. Nat Rev Mol Cell Biol.

[15] Ertürk A, Bradke F (2013). High-resolution imaging of entire organs by 3-dimensional imaging of solvent cleared organs (3DISCO). Experimental neurology.

[16] Fehrenbach, J., Kovac, B. & Weiss, P. FitEllipsoid: a fast supervised ellipsoid segmentation plugin. Preprint at https://hal.archives-ouvertes.fr/hal-01520337 (2017). 10.1186/s12859-019-2673-0 PMC6419800 30876406

[17] Lazaro-Dieguez F, Ispolatov I, Musch A (2015). Cell shape impacts on the positioning of the mitotic spindle with respect to the substratum. Molecular biology of the cell.

[18] Thery M (2005). The extracellular matrix guides the orientation of the cell division axis. Nat Cell Biol.

[19] Williams SE, Beronja S, Pasolli HA, Fuchs E (2011). Asymmetric cell divisions promote Notch-dependent epidermal differentiation. Nature.

[20] Versaevel M, Grevesse T, Gabriele S (2012). Spatial coordination between cell and nuclear shape within micropatterned endothelial cells. Nature communications.

[21] Carreno S (2008). Moesin and its activating kinase Slik are required for cortical stability and microtubule organization in mitotic cells. J Cell Biol.

[22] Lancaster OM (2013). Mitotic rounding alters cell geometry to ensure efficient bipolar spindle formation. Developmental cell.

[23] Magidson V (2011). The spatial arrangement of chromosomes during prometaphase facilitates spindle assembly. Cell.

[24] Butcher DT, Alliston T, Weaver VM (2009). A tense situation: forcing tumour progression. Nat Rev Cancer.

[25] Northey JJ, Przybyla L, Weaver VM (2017). Tissue Force Programs Cell Fate and Tumor Aggression. Cancer discovery.

[26] Huisken J, Stainier DY (2009). Selective plane illumination microscopy techniques in developmental biology. Development.

[27] di Pietro F, Echard A, Morin X (2016). Regulation of mitotic spindle orientation: an integrated view. EMBO reports.

[28] Legoff L, Rouault H, Lecuit T (2013). A global pattern of mechanical stress polarizes cell divisions and cell shape in the growing Drosophila wing disc. Development.

[29] Wyatt T, Baum B, Charras G (2016). A question of time: tissue adaptation to mechanical forces. Curr Opin Cell Biol.

[30] Thery M, Jimenez-Dalmaroni A, Racine V, Bornens M, Julicher F (2007). Experimental and theoretical study of mitotic spindle orientation. Nature.

[31] Wyatt TP (2015). Emergence of homeostatic epithelial packing and stress dissipation through divisions oriented along the long cell axis. Proc Natl Acad Sci USA.

[32] Gibson WT (2011). Control of the mitotic cleavage plane by local epithelial topology. Cell.

[33] Minc N, Burgess D, Chang F (2011). Influence of cell geometry on division-plane positioning. Cell.

[34] Cordeiro MH, Smith RJ, Saurin AT (2018). A fine balancing act: A delicate kinase-phosphatase equilibrium that protects against chromosomal instability and cancer. The international journal of biochemistry & cell biology.

[35] Musacchio A, Salmon ED (2007). The spindle-assembly checkpoint in space and time. Nat Rev Mol Cell Biol.

[36] Heald R, Khodjakov A (2015). Thirty years of search and capture: The complex simplicity of mitotic spindle assembly. J Cell Biol.

[37] Noatynska A, Gotta M, Meraldi P (2012). Mitotic spindle (DIS)orientation and DISease: cause or consequence?. J Cell Biol.

[38] Pease JC, Tirnauer JS (2011). Mitotic spindle misorientation in cancer–out of alignment and into the fire. J Cell Sci.

[39] Laurent, J. F. *et al*, V. Multicellular tumor spheroid models to explore cell cycle checkpoints in 3D. *BMC Cancer* 13 (2013). 10.1186/1471-2407-13-73 PMC3598667 23394599

[40] de Chaumont F (2012). Icy: an open bioimage informatics platform for extended reproducible research. Nat Methods.

[41] Schindelin J (2012). Fiji: an open-source platform for biological-image analysis. Nat Methods.

