# Supplementary material for: Impact of physical confinement on nuclei geometry and cell division dynamics in 3D spheroids
Source: Sci Rep. 2018 Jun 8;8:8785. doi: 10.1038/s41598-018-27060-6 (PMC5993719; doi:10.1038/s41598-018-27060-6)
Supplement: Supplementary file 1 — Supplementary information [file 41598_2018_27060_MOESM1_ESM.pdf]

Impact of physical confinement on nuclei geometry and cell division dynamics in 3D spheroids

Annaïck DESMAISON[1°], Ludivine GUILLAUME[1°], Sarah TRICLIN[1], Pierre WEISS[1,2], Bernard DUCOMMUN[1,3] and Valérie LOBJOIS[1*]

°: AD and LG equally contributed to this work.

Affiliations:

[1] ITAV, Université de Toulouse, CNRS, Toulouse, France

[2] IMT, Université de Toulouse, CNRS, Toulouse, France

[3] CHU de Toulouse, Toulouse, France

***Corresponding author:**

Dr Valérie LOBJOIS

Centre Pierre Potier, ITAV – USR3505,

1 place Pierre Potier

31106 Toulouse Cedex 1, France

valerie.lobjois@itav.fr

Supplementary Figures

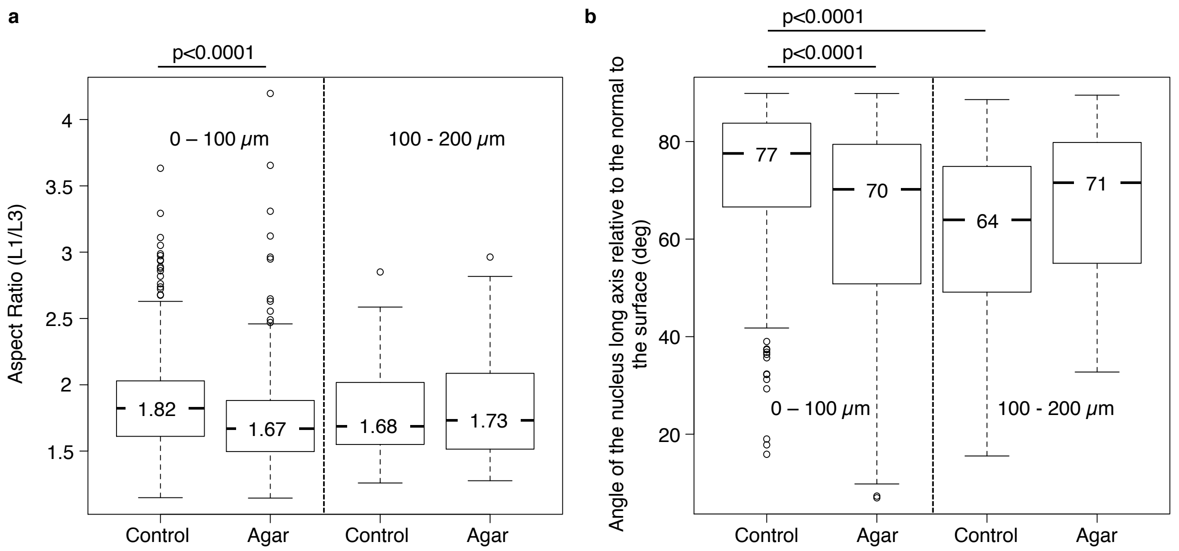

Supplementary Figure S1: Analysis of nucleus geometry in depth in spheroids
(a) Boxplots (R software) of the L1 to L3 ratio for nuclei located between 0 and 100 µm (left), and between 100 and 200 µm (right) from the surface in control spheroids (control) and spheroids grown in 1% low-melting point agarose for 24 hours (Agar) before fixation. A high ratio value indicates that the nucleus is elongated; n= 327 and 479 nuclei from control and agarose-embedded spheroids, respectively (6 to 13 independent spheroids were analysed in each condition). (b) Boxplots showing the orientation of nuclei located between 0 and 100 µm (left), and between 100 and 200 µm (right) from the surface in control and agarose-embedded (Agar) spheroids. Only nuclei with an L1/L3 value higher than 1.5 (and thus considered to be elongated) were analysed. A 90° angle means that L1 is parallel to the spheroid convex hull. The aspect ratio and the angle values are significantly different in control and in agarose-embedded spheroids ($p<0.0001$ in both case). The median values are indicated in the boxes.

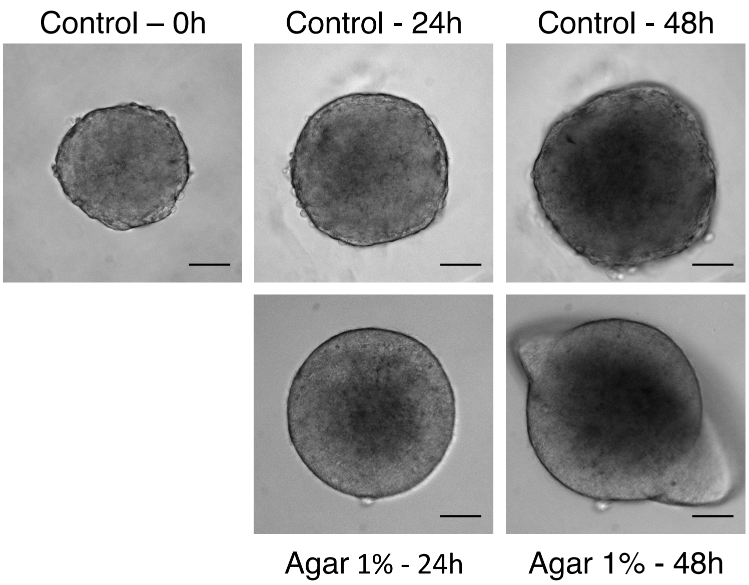

Supplementary Figure S2: Growth of agarose-embedded spheroids.
Transmitted-light images of spheroids grown in suspension (Control) and spheroids confined in 1% low-melting agarose (Agar) for 24 hours or 48 hours. No space is visible between the spheroid and the surrounding agarose. The shape modification of the spheroid due to the agarose rupture is clearly visible at 48 hours. Scale bar: 100 µm.

Legends to supplementary movies

Supplementary movie 1: Live 3D light-sheet fluorescence microscopy (LSFM) imaging of a spheroid.

This movie shows the maximal projection of an LSFM stack (130 frames) of a spheroid made of HCT116 cells that express histone H2B-mCherry and grown in suspension. Cells undergoing mitosis during the experiment are indicated by arrows.

Supplementary movie 2: Live 3D light-sheet fluorescence microscopy (LSFM) imaging of a mitotic cell in a spheroid.

This movie shows one dividing cell within a spheroid of HCT116 cells that express histone H2B-mCherry. Left: fluorescence corresponding to the mCherry signal. Right: The red surface corresponds to the segmentation performed with the Imaris software.

Supplementary movie 3: Live imaging of an HCT116 spheroid after confinement in agarose.

Transmitted light time-lapse acquisition of an HCT116 spheroid after embedding in 1% low-melting agarose.

Supplementary movie 4: Live imaging of the mitosis steps in a spheroid.

3D visualization of a small region of a spheroid made of HCT116 cells that express histone H2B-mCherry grown in suspension. Images were acquired by LSFM and processed using the Imaris software. In this 1.5-hour movie, two cells are progressing through mitosis (white arrows).

Supplementary movie 5: Live imaging of mitosis in an agarose-confined spheroid

3D visualization of a small region of a spheroid of HCT116 cells that express histone H2B-mCherry grown in agarose for 24 hours. Images were acquired by LSFM and processed using the Imaris software. In this 2-hours movie, one cell is progressing through mitosis.

Supplementary movie 6: Metaphase plate in a spheroid

3D representation of a mitotic cell within a spheroid of HCT116 cells that express histone H2B-mCherry grown in suspension for 24 hours. Chromosomes are aligned on the metaphase plate, organized in a thin and fully filled cylinder. The image stack was acquired by LSFM and processed using Imaris.

Supplementary movie 7: Metaphase plate in a confined spheroid

3D representation of a mitotic cell within a spheroid of HCT116 cells that express histone H2B-mCherry embedded in agarose without any treatment for 24 hours. Mitotic condensed chromosomes are organized in a broad ring, with a chromosome-free lumen at the centre. The image stack was acquired by LSFM and processed using Imaris.
